# No correlation between microbiota composition and blood parameters in nesting flatback turtles (*Natator depressus*)

**DOI:** 10.1038/s41598-020-65321-5

**Published:** 2020-05-20

**Authors:** T. Franciscus Scheelings, Robert J. Moore, Thi Thu Hao Van, Marcel Klaassen, Richard D. Reina

**Affiliations:** 1 School of Biological Sciences, Monash University, Wellington Rd, Clayton, Victoria 3800 Australia; 2 School of Science, RMIT University, Bundoora West Campus, Plenty Rd, Bundoora, Victoria 3083 Australia; 3 Centre for Integrative Ecology, Deakin University, Waurn Ponds, Victoria 3216 Australia

**Keywords:** Biochemistry, Microbiome

## Abstract

The microbiota is considered critical for normal vertebrate homeostasis and it may exert its effects at a local level within the gastrointestinal tract, or systemically through the production of bacterial metabolites. To date, investigations into the role that the microbiota plays in reptile physiology are rare. To address this knowledge gap, we explored the relationship between differences in microbial communities to see if they accounted for differences in haematology and biochemistry values, in different populations of nesting flatback turtles (*Natator depressus*). We found that microbiota composition was not correlated to any of the blood analytes we measured in flatbacks. This study is the first of its kind in reptiles and highlights the need for further investigations to determine mechanisms by which the microbiota influences the physiology and health of reptiles.

## Introduction

Diverse and complex communities of microorganisms, known collectively as the microbiota, reside on and within every metazoan species. The occupation of higher organisms by prokaryotic and eukaryotic colonists is thought to be a key factor in driving evolution and radiation of life on Earth^1,2^. Primarily based on human research, with a limited number of investigations in other animals, we are only just beginning to understand the complexity of the host-microbiota relationship, and how it contributes to normal physiology and homeostasis^3^. For example, the microbiota communicates with adipocytes thus influencing obesity and insulin resistance, as well as browning (important for thermoregulation) and inflammation^4^. It plays a role in hepatic function by modulating bile acid metabolism, lipogenesis and energy expenditure^4^. It has an effect on insulin secretion from the pancreas and shapes whole-body growth^4^. Interestingly, the microbiota has been shown to play an important role in changing behaviour by affecting serotonin metabolism, influencing intestinal gluconeogenesis, impacting the permeability of the blood-brain barrier, and regulating appetite^4^. Importantly, the microbiota also primes and directs maturation of the immune system^5^, and contributes to the function of both the innate and adaptive immune systems^1,6^. In comparison to other vertebrates, the reptile immune system is relatively poorly understood. Like all jawed vertebrates, reptiles possess both an innate and adaptive immune system^7^ and they also possess many of the anatomical and cellular components that higher vertebrates, such as birds and mammals, use in defending themselves from invading pathogens^7^. Given these similarities, it may be speculated that the microbiota of reptiles has a similar effect on their immune system maturation and function as is seen in other vertebrates.

To date, most microbiota studies in sea turtles have focused on descriptions of community composition^8–11^, with some investigations describing changes in the microbiota in response to age and habitat use^12,13^, as well as in health and rehabilitation^14,15^. However, to our knowledge, there have been no attempts to link microbiota composition with specific blood parameters in sea turtles. In an endeavour to correct this deficit, we explored whether differences in microbiota composition correlated with differences in haematology and biochemistry results in two populations of nesting flatback turtles (*Natator depressus*). Listed as ‘Data Deficient’ by the IUCN^16^, we know relatively little about this species but its restricted range and relatively small population size make it a useful study species. It is the single member of the genus *Natator*, and is one of only two marine turtle species that does not have a global distribution^17^. All recorded flatback nesting beaches occur in Australia^18^ and the species feeds widely throughout the waters of the Australian continental shelf^17^. Four major management units have been identified for flatback turtle rookeries in Australia^17^, with genetic analysis indicating that there is a low level of genetic variability in the species and there is limited gene flow between the rookeries^19^.

As an indicator of immune system function, we investigated the relationships between microbial composition and red and white blood cell indices. To assess general organ function we examined how microbiota correlated with a range of biochemical parameters including aspartate aminotransferase, uric acid, creatinine kinase, total protein, glucose, calcium, phosphate, albumin, globulin, potassium, and sodium. We chose these analytes because they are recommended analytes for the assessment of health in chelonians^20^, and are able to be measured by the Vet Scan analyzer (Abaxis, Union City, California, USA). We sampled animals from two spatially separate populations because environmental factors have been shown to influence both blood values^21–23^, and microbiota composition^24–26^ of wild animals. Thus, sampling animals from different populations was important to elucidate if any consistent correlations exist between blood analytes and microbiota.

Alterations in the microbiota have been shown to affect the fitness of humans and other species^27^, although the implications for these variations in an ecological and conservation context are poorly understood. Improving our comprehension of the host-microbial relationship, and factors that drive dysbiosis and reduced fitness, are imperatives for modern conservation efforts^27^. This information may be used as a screening tool to monitor how animals are able to respond, in a physiological sense, to an ever-changing world. This is especially important for sea turtles, which as a group are some of the most imperilled species on Earth^23^.

Exploring the effects of microbiota on physiology and health in wild animals is difficult, due to the multitude of confounding factors that cannot be controlled for. These may include genetic differences between populations, environmental health, and climactic conditions. Given that there is little genetic variation between groups of flatback turtles, and on a global scale they occupy a relatively small ecological niche, we felt that they would make an ideal model species in which to study the effects of microbial composition on health indices in wild animals for which housing in a controlled laboratory environment was not possible or practical. Although the flatback turtle is the only member of the genus *Natator*, with the exception of the leatherback turtle (*Dermochelys coriacea*), it still shares a close phylogenetic relationship to all other extant species of sea turtles^28^, and thus the results of this investigation are likely to be applicable to a range of species. Our aims were to determine and compare the microbiota and blood parameters of two flatback populations and to detect measurable relationships between the microbiota and the health of animals.

## Methods

### Ethics statement

This study was approved by the Biological Sciences Animal Ethics Committee of Monash University (approval 14694), and all experiments were performed in accordance with relevant guidelines and regulations. Adult flatback turtles (*Natator depressus*) were sampled under permit WITK17730216 from the Queensland Department of Environment and Heritage Protection, and the permit 01-000121-1 from the Department of Parks and Wildlife Western Australia.

### Study population

Nesting flatback turtles were sampled from Crab Island, Queensland, Australia (10.9947° S, 142.1090° E) in September 2016 (n = 20), and from Port Hedland, Western Australia, (20.3107° S, 118.5878° E) in November 2016 (n = 17).

### Sample collection

During the nesting season female flatback turtles came ashore to dig a nest and lay eggs, at which time we were able to collect samples when females had finished nesting and were returning to the ocean. For blood collection, an area of skin was prepared using alcohol wipes and 10 ml of blood was collected from the dorsal cervical sinus using an 18 G needle attached to a 10 ml syringe. The turtle was then flipped into dorsal recumbency, and an equine uterine swab (Minitube, Smythesdale, Victoria, Australia) was inserted into the cloaca so that it entered the distal colon. These swabs are approximately 90 cm in length and each swab was inserted to a depth of at least 60 cm. Based on previous research conducted by our research group, in which endoscopy was used to visualise the cloacal structures of sea turtles^29^, we are confident that inserting the swabs to this depth permitted sampling of the distal colon and not the cloaca. The swabs were housed in a sterile sheath, the entire apparatus was inserted into the cloaca and the swab tip was extruded when correct placement of the sheath had been achieved within the colon. The swab tip was then retracted back into the sheath prior to extraction. The tip of the swab was cut using a sterile wire cutter, placed into a sterile Eppendorf tube without buffer, and sealed. Turtles were then permitted to return to the ocean. Total sample collection time was approximately 10 minutes. The Eppendorf tube containing the swab was immediately placed into a portable cool box filled with ice, and once back at the field station they were frozen at −20 °C for approximately 3–4 days. Swabs were transported back to the laboratory using dry ice (−78.5 °C), where they were stored at −80 °C until extraction could take place approximately 1 week later.

### Analysis of blood

Immediately after blood had been collected, one milliliter was transferred into a lithium heparin container (BD Microtainer tubes, Becton Dickinson, Franklin Lakes, New Jersey, USA) and the remainder into plain BD Microtainer Tubes (Becton Dickinson). At this same time a drop of fresh blood (without anticoagulant) was placed onto a microscope slide, smeared using the beveled edge of another slide and allowed to air-dry. The blood tubes were then placed into a portable ice pack and taken back to the field laboratory. Once at the field laboratory, the packed cell volume (PCV) was determined using standard centrifugation in microhematocrit tubes and the microscope slides were stained with Romanowsky stain (Rapid Diff, Australian Biostain Pty. Ltd., Traralgon, Victoria, Australia). Leukocyte differential counts were performed manually on blood films, and white cells were classified as heterophils, lymphocytes, eosinophils, basophils, or monocytes^30^. Heterophil/eosinophil counts were performed manually using a hemocytometer and by staining whole blood with phloxine B (made in-house). The total white blood cell (TWBC) count was calculated by correcting the manual count for the percentage of heterophils and eosinophils present^31^. In addition, we calculated the heterophil/lymphocyte ratio (H:L) as a measure of stress^32^. Both PCV and TWBC counts were determined within 3 h of blood collection. Blood in the plain tube was centrifuged, and the resultant serum removed and stored in a portable freezer (−20 °C) in the field for a maximum of three days. The serum was then transported to the lab on dry ice and frozen at −70 °C for up to 2 months until analysis. Serum was analyzed using the avian-reptilian rotor on the Vet Scan analyzer (Abaxis, Union City, California, USA). Parameters able to be measured using the Vet Scan analyser include aspartate aminotransferase (AST), uric acid (UA), creatinine kinase (CK), total protein (TP), glucose (Glu), calcium (Ca^2+^), phosphate (Phos), albumin (Alb), globulin (Glob), potassium (K^+^), and sodium (Na^+^).

### DNA extraction of cloacal swabs

DNA was extracted manually using the phenol-chloroform method^33^. In each Eppendorf tube, 500 μL of extraction buffer (20 mM ethylenediaminetetraacetic acid (EDTA), 0.1 M Tris, 1% cetrimonium bromide, 56 mM NaCl, pH 8) was added so that swabs were completely covered. We then added 20 μL of proteinase K (Qiagen proteinase K (10 ml) to each vial, along with 60 μL of 10% sodium dodecyl sulphate. The mixture was then incubated at 55 °C overnight. The next day, 50 μL of 5 M NaCl and 500 μL of phenol was added, and the tubes shaken until an emulsion was formed. They were then incubated at room temperature for 10 minutes, with intermittent mixing. The tubes were then centrifuged at 10,000 RPM for 10 minutes and the supernatant removed and added to a new tube containing 250 μL phenol and 250 μL chloroform:isoamyl-alcohol (24:1). The tubes were again centrifuged at 10,000 RPM for 10 minutes and the resultant supernatant added to a new tube containing 500 μL of chlorophorm:isoamyl-alcohol. Once again, the tubes were centrifuged at 10,000 RPM for 10 minutes. The supernatant was then added to a new tube containing 3 M Sodium Acetate at a volume equal to 10% of the extraction solution. We then added 1 ml of ice-cold 99% ethanol to each test tube and then placed them into a freezer at −20 °C for 1 hour. The tubes were then centrifuged at 4 °C at 12,000 RPM for 10 minutes. The fluid in the test tube was then removed with a glass pipette and 1 ml of ice-cold 70% alcohol was added. The tubes were centrifuged a final time at 4 °C at 12,000 RPM for 5 minutes. After centrifugation the alcohol was removed and the lids left off the tubes to allow the DNA pellet to dry. Once dried, 25 μL of 1 x Tris-EDTA (TE) was added to each tube and the extracted DNA was stored at −20 °C until amplicon sequencing could take place.

### rRNA gene amplicon sequencing

The V3-V4 region of 16 S rRNA genes were amplified with forward primer 5′ ACTCCTACGGGAGGCAGCAG 3′ and reverse primer 5′ GGACTACHVGGGTWTCTAAT 3′ using Q5 high fidelity polymerase (New England Biolabs) using the barcoding strategy of Fadrosh, *et al*.^34^. Sequencing was performed on an Illumina MiSeq system (2 × 300 bp).

### Data processing

Sequence data was analysed using QIIME version 1.9.1^35^ using default parameters and a Phred quality threshold of >20. The UCLUST algorithm^36^ was used to pick OTUs at 97% sequence identity and a Biome table was produced. Potentially chimeric sequences were identified using Pintail^37^. BLAST was used to assign taxonomy against the Greengenes database^38^ and QIIME version 1.9.1 defaults. Additional assignment of taxonomy was performed using a command line version of BLASTN^39^ against the NCBI 16S Microbial database.

### Statistics and data analysis

For blood results, statistical analysis was performed using the statistical software program R (R Development Core Team 2015). For all data collected, ranges were calculated by Dixon Q test analysis of data with outliers (defined by a D/R ratio greater than 1:3) excluded. Data were assessed for normality with the Shapiro-Wilk test. For normally distributed data, Welch’s two sample t-test was conducted to identify whether there was a significant difference in hematologic and biochemical values between populations, and for non-normally distributed data we used the Wilcoxon Rank sum test. Significance was accepted at p < 0.05.

Microbiota data was rarefied to a depth of 3,000 sequence reads and initial exploration of the Biome table data was performed using the online Calypso software (http://cgenome.net/wiki/index.php/Calypso)^40^. Data was further analysed in R, utilising the package ‘phyloseq’^41^. Alpha diversity (what species are present and how many different types of species are present with a sample) was explored using Observed OTUs (the number of different OTUs seen), Shannon index (a measure of how evenly microbes are distributed within a sample) and Chao1 (a measure of the predicted number of OTUs within a sample, given that there is a finite number of OTUs in that community) estimates. Alpha diversity was tested for normality using the Shapiro-Wilks test and then parametric and non-parametric methods were adopted to test for significant differences between groups. Both Observed OTUs (W = 0.89, p < 0.01) and Shannon diversity (W = 0.85, p < 0.01), were not normally distributed, so comparisons were made using the Kruskal-Wallis test. Chao1(W = 0.97, p = 0.13), was normally distributed and so comparisons between populations were made using Welch’s two sample t-test. Beta diversity (how similar or different are samples) was investigated using non-metric multidimensional scaling (NMDS) (Bray-Curtis) and Adonis tests. The variance of each group plotted by the NMDS analysis was tested with multivariate homogeneity of group dispersion.

To determine if microbiota samples was correlated to haematological and biochemical data we combined individual blood parameter results with the microbiota NDMS coordinates (NDMS1 and NDMS2) for each turtle. Each blood parameter was analysed independently against the microbiota composition for the entire population of each locality. We confirmed the suitability of this data for linear model testing by exploring the homogeneity of variances (residuals vs fitted plots and scale-location plots), the normality of residuals (Q-Q plots), and Cook’s Distance. Although some relationships had the appearance of being non-monotonic when fitted with a loess smoothed line (Supplementary Fig. 1), an examination of the diagnostic plots still supported a best interpretation that the residuals of all model(s) were suitable for linear fitting. OTUs were subjected to a similar screening prior to investigating their relationships with the two NMDS coordinates (see below). Because the interaction of population and NMDS coordinates was of interest, linear models included the main effects population and NMDS coordinate, as well as the interaction term site:NMDS (i.e. each blood parameter was tested as a function of NMDS1 + population + NMDS1 x population, and this was repeated for NMDS2). Given the high number of repeated tests we elected to use a significance threshold of p = 0.01 to help control for familywise Type I error.

## Results

### Blood results

We found significant differences between the two populations for PCV (t = 4.02, df = 32.96, p < 0.01), heterophils (t = −3.01, df = 34.09, p = 0.01), heterophil/lymphocyte ratio (H:L) (W = 90, p = 0.02), basophils (W = 241, p < 0.01), uric acid (t = −6.28, df = 27.85, p < 0.01), total protein (W = 292.5, p < 0.01), globulin (W = 278, p < 0.01), albumin (W = 81, p < 0.01) and potassium (W = 283, p < 0.01) (Table 1).

**Table 1:** Comparison of hematology and serum biochemistry results for free-ranging nesting flatback turtles (*Natator depressus*) captured at Crab Island, September 2016, and Port Hedland, October 2016.

Parameter	Crab Island (*n* = 20)		Port Hedland (*n* = 17)		p	t	df	W
	Range	Mean ± SE	Range	Mean ± SE				
PCV (%)	24.0–38.0	30.9 ± 4.1	29.0–43.0	36.5 ± 4.4	<0.01	4.03	32.96	N/A
TWBC (10^3^/µL)	3.8–13	7.9 ± 3.3	3.0–11.1	6.5 ± 2.6	0.29	N/A	N/A	135
Heterophils (10^3^/µL)	2.2–8.5	4.4 ± 1.7	1.0–5.6	2.9 ± 1.2	0.01	−3.01	34.09	N/A
Lymphocytes (10^3^/µL)	0.6–5.7	2.6 ± 1.6	0.8–6.0	2.7 ± 1.7	0.80	N/A	N/A	179
H:L	0.8–4.8	2.2 ± 1.2	0.6–3.0	1.4 ± 0.7	0.02	N/A	N/A	90
Monocytes (10^3^/µL)	0.0–0.7	0.2 ± 0.2	0.0–0.7	0.2 ± 0.2	0.94	N/A	N/A	167
Eosinophils (10^3^/µL)	0.2–1.8	0.6 ± 0.4	0.0–1.4	0.1 ± 0.1	0.64	N/A	N/A	154
Basophils (10^3^/µL)	0.0–0.4	0.1 ± 0.1	0.0–0.4	0.1 ± 0.1	0.03	N/A	N/A	241
Aspartate aminotransferase (U/L)	94.0–493.0	184.2 ± 100.8	92.0–317.0	185.8 ± 77.3	0.90	N/A	N/A	174.5
Creatinine kinase (U/L)	182.0–1464.0	527.7 ± 360.6	111.1–1040.0	455.1–302.9	0.40	N/A	N/A	142
Uric acid (µmol/L)	24.0–111.0	59.0 ± 24.5	5.0–51.0	20.4–11.5	<0.01	−6.28	27.85	N/A
Glucose (mmol/L)	3.5–6.3	4.7 ± 0.8	3.5–5.6	4.4 ± 0.5	0.11	−1.64	33.4	N/A
Calcium (mmol/L)	0.9–5.0	2.9 ± 0.8	0.5–5.0	2.7 ± 1.4	0.87	N/A	N/A	164
Phosphorus (mmol/L)	2.4–4.5	3.0 ± 0.5	2.1–7.0	3.3 ± 1.2	0.88	N/A	N/A	175.5
Total protein (g/L)	26.0–47.0	34.8 ± 6.3	33.0–78.0	46.0 ± 9.9	<0.01	N/A	N/A	292.5
Albumin (g/L)	10.0–25.0	15.4 ± 4.4	13.0–37.0	21.8 ± 5.1	<0.01	N/A	N/A	281
Globulin (g/L)	15.0–26.0	19.3 ± 3.4	19.0–40.0	24.3 ± 5.4	<0.01	N/A	N/A	278
Potassium (mmol/L)	5.0–8.2	6.4 ± 0.7	6.2–12.0	7.9 ± 1.9	<0.01	N/A	N/A	283
Sodium (mmol/L)	143.0–159.0	149.7 ± 4.5	142.0–160.0	149.2 ± 5.1	0.68	−0.3	32.26	N/A

Analytes were tested for normality using the Shapiro-Wilk test and then parametric and non-parametric methods were used to test for differences between locality.

### Microbiota composition

**Figure 1:**
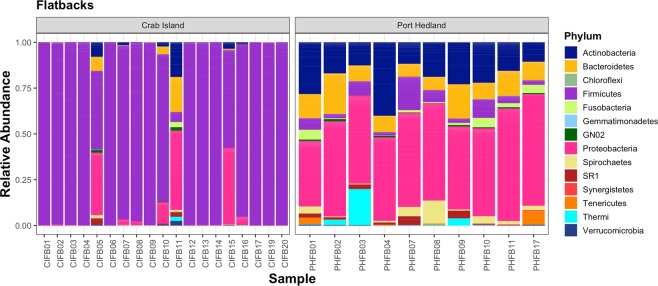
Relative abundance of microbial phyla in each flatback turtle group, CI and PH, show significant differences between the two populations. Firmicutes was far more abundant in Crab Island animals compared to Port Hedland animals, while Actinobacteria and Proteobacteria were more abundant in Port Hedland animals compared to Crab Island animals.

**Figure 2:**
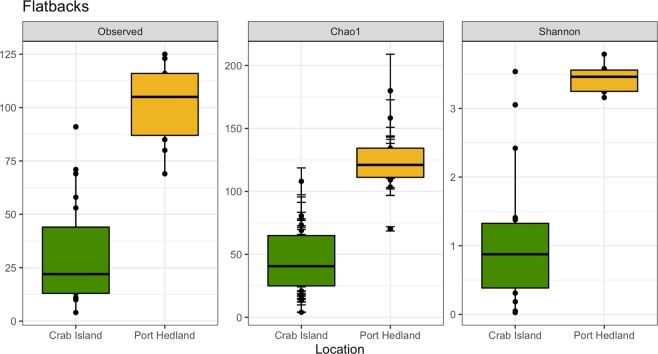
Alpha diversity estimates for gut microbial communities for flatback turtles from Crab Island, Queensland and Port Hedland, Western Australia. Individual points and brackets represent the richness estimate and the theoretical standard error range associated with that estimate, respectively. Within each panel, the samples are organized into location of capture, and a boxplot is overlaid on top of this for the two groups. Significant differences existed for all alpha diversity metrics, Observed OTUs (χ^2^ = 17.26, df = 1, p < 0.01), Shannon diversity (χ^2^ = 16.30, df = 1, p < 0.01), and Chao1 estimates (t = −7.05, df = 16.97, p < 0.01).

**Figure 3:**
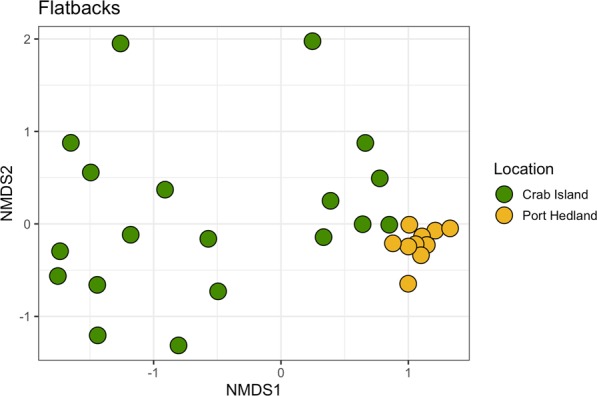
NMDS plot of Bray-Curtis distances for gut microbiota in flatback turtles from two locations, Crab Island, and Port Hedland. Each point represents the gut microbiota of an individual turtle. Observed differences between the two populations were significant (Adonis R^2^ = 0.27, p < 0.01).

After rarefaction, 16S rRNA gene sequence were able to be included from 19 animals from Crab Island and from 10 animals from Port Hedland. In total, we identified 274 operational taxonomic units (OTUs) (Supplementary Table S1). Within the microbiota, *Firmicutes* was by far the most predominate phylum in animals from Crab Island, while *Proteobacteria* was the most commonly identified phylum in Port Hedland turtles, followed by *Actinobacteria*, *Bacteroidetes* and *Firmicutes* (Fig. 1). Microbial diversity differed markedly among flatback turtle groups for all Alpha diversity metrics, Observed OTUs (χ^2^ = 17.26, df = 1, p < 0.01), Shannon diversity (χ^2^ = 16.30, df = 1, p < 0.01), and Chao1 estimates (t = −7.05, df = 16.97, p < 0.01) (Fig. 2). NMDS analysis of microbiota composition indicated that animals from Port Hedland clustered closely together, with similar overall composition, while animals from Crab Island had a more diverse pattern (Fig. 3). The observed differences between populations were statistically significant (df = 1, SS_T_ = 2.84, MS_T_ = 2.84, f = 10.2, R^2^ = 0.27, p < 0.01). Beta dispersion analysis of the NMDS plot revealed that there were significant differences in variance between microbiota composition for the two locations (df = 1, SS_T_ = 0.19, MS_T_ = 0.19, f = 5.64, p = 0.03). Overall, 26 (28.2%) of the OTUs were shared between the two populations (Supplementary Table S2). Sixty-one (66.3%), OTUs were unique to animals from Port Hedland, in comparison to 5 (5.4%) unique OTUs from animals from Crab Island (Supplementary Table S2).

### Correlation of blood and microbiota results

No correlation was found between microbiota composition and any of the blood parameters analysed in this investigation PCV (NMDS1 p = 0.82, NMDS2 p = 0.71); TWBC (NMDS1 p = 0.14, NMDS2 p = 0.99), lymphocytes (NMDS1 p = 0.22, NMDS2 p = 0.79), heterophils (NMDS1 p = 0.14, NMDS2 p = 0.95); heterophil:lymphocyte ratio (NMDS1 p = 0.47, NMDS2 p = 0.71); monocytes (NMDS1 p = 0.61, NMDS2 p = 0.46), eosinophils (NMDS1 p = 0.96, NMDS2 p = 0.60), basophils (NMDS1 p = 0.05, NMDS2 p = 0.46); aspartate aminotransferase (NMDS1 p = 0.64, NMDS2 p = 0.68), creatinine kinase (NMDS1 p = 0.74, NMDS2 p = 0.53), uric acid (NMDS1 p = 0.71, NMDS2 p = 0.70); glucose (NMDS1 p = 0.81, NMDS2 p = 0.24), calcium (NMDS1 p = 0.62, NMDS2 p = 0.86); phosphate (NMDS1 p = 0.82, NMDS2 p = 0.34), total protein (NMDS1 p = 0.37, NMDS2 p = 0.72); albumin (NMDS1 p = 0.09, NMDS2 p = 0.9); globulin (NMDS1 p = 0.86, NMDS2 p = 0.67); sodium (NMDS1 p = 0.09, NMDS2 p = 0.83); and potassium (NMDS1 p = 0.29, NMDS2 p = 0.39) (Supplementary Fig. S1, Supplementary Table S3).

## Discussion

Despite demonstrating significant differences in blood parameters and in microbiota composition, in separate populations of the same sea turtle species, we did not detect any observed differences that were mediated by host-microbiota interactions, or by other extraneous factors. While we did not demonstrate blood parameters in these animals that were influenced by bacterial community structure, our results need to be interpreted with the knowledge that these experiments were not performed in a controlled laboratory setting. Housing adult sea turtles in a captive environment in sufficient numbers to perform such an investigation is impossible. Furthermore, captivity has been shown to markedly affect the microbiota of a range of non-domestic species^42–49^, and so this would be a major confounding factor for any manipulative study involving captive wildlife. In a similar manner, under certain circumstances, manipulative investigations using human subjects maybe impractical or unethical and so a range of disparate vertebrate species are used to model specific conditions, from which human physiological responses are inferred^50,51^. Therefore, it is important to note that throughout the discussion we offer plausible accounts for any observed differences in parameters based on available literature in multiple species, but we recognise that they may not be definitive explanations for our experimental results. Importantly, we are unable to say categorically that any observed correlation between microbiota and blood parameter is as a result of host-microbiota, or microbiota-host interactions. Investigations such as ours that focus on uncommon, or difficult to access species, in wild environments, are important for advancing our understanding of the microbiota and the role that it has played in shaping vertebrate evolution.

In this study, we discovered a significant difference for PCV between the two populations of flatback turtles. In mammals, PCV has been linked to microbiota composition, particularly during periods of inappetence, such as during hibernation. At these times there is a decrease of expression of the rate-limiting enzyme of bile acid production CYP7A1 in the liver of hibernating animals^52–54^, and given that the microbiota contributes to bile acid production, this decreased bile acid regulation may result in a relative increase in circulating bile acids^55^. Certain bile acids, such as deoxycholic acid, and lithocholic acid, both of which are dependent on the microbiota, are known to have haemolytic activity, which may result in destruction of red blood cells, thus lowering PCV^54^. Similarly, sea turtles undergo periods of complete or reduced fasting during the breeding and nesting phases of their life^56^, which may result in a concurrent rise in circulating bile acid concentration within the blood. Therefore, it may be expected that the differences in microbiota observed between the two populations may have influenced bile acid production, and as a corollary, PCV in this study. However, we were not able to demonstrate any measurable relationship between microbiota and PCV in this investigation. Furthermore, we were not able to measure bile acids to determine if differences in microbiota resulted in differences in bile acid levels, nor were we able to assess if there were differential foraging efforts between animals of the two populations during nesting. Additionally, no data exists for PCV levels of foraging flatback turtles, so we were unable to determine if the PCV in our results were different than would be expected in non-breeding turtles. In the absence of microbiota exerting any effect on PCV, the most likely explanation for this observation is due to differences in hydration. In comparison to Crab Island animals, Port Hedland flatbacks had a relative increase in PCV and total protein, which may be an indication of mild dehydration in these animals^57^. In humans, the microbiota has been shown to influence the cellular transport of solutes through the gut mucosa and contribute to the hydration of individuals, due to changes in plasma osmolality^58^.

During this study we noted a significant difference in total protein, albumin, and globulin between the two groups of turtles. Protein levels in reptiles may change in response to hydration, vittelogenesis in females, inflammation, liver, kidney and gastrointestinal function, season, and feeding or fasting status^20^. In humans suffering from primary biliary cirrhosis, alterations in the microbiota are implicated in changes to total protein and globulin, as well as a range of liver-specific enzymes^59^. There was no evidence that any of the turtles in either of our study sites were obviously unwell, but we did not perform any liver-specific functional testing. The role that the microbiota plays in hepatic protein synthesis and metabolism in sea turtles remains unknown. Another possible explanation for differences in protein levels may relate to periods of inappetence. Flatback turtles from Western Australia undergo a shorter migration to their natal beaches than Crab Island turtles, as they forage relatively close to their nesting rookeries^60^. This might mean that turtles from Crab Island are inappetant for a longer period of time, and therefore will undergo greater protein catabolism in an effort to fuel their migration, thus resulting in relatively lower serum protein levels.

Related to the observed alterations in serum proteins in this study, was the differences in uric acid concentration. The role that both the small and large intestinal microbes play in dietary protein metabolism are well known^61^. Secondary metabolites from bacterial degradation of peptides is dependent on the type of proteins ingested, as well as the composition of the intestinal microbial community^61^. In chelonians, the end product of purine metabolism may be a combination of ammonia, urea and uric acid, with the proportion of each metabolite -dependent on species and life history^62,63^. Hyperuricaemia is associated with alteration of the gut microbiota in mice^64^, but we did not demonstrate that differences in microbial communities between flatback turtle populations accounted for differences in uric acid levels. We did not assess microbial function and protein metabolism as a component of this study, but the role that the microbiota plays in protein degradation in sea turtles warrants further investigation, and may also explain the correlations that we observed with total protein, albumin, and globulin.

Hyperkalaemia is commonly associated with chronic renal disease in humans, and may be exacerbated by alterations in the microbiome^65^. We did not see a correlation between microbiome and potassium levels in flatback turtles, but assessing renal function in chelonians is difficult without the use of endoscopic biopsies^66^ or other techniques beyond the scope of this study. The blood parameters reported in this investigation are the first reported for the flatback turtle and determining what is normal for blood potassium levels is difficult, so it may be that animals from Crab Island are hypokalaemic.

We found that absolute heterophil and basophil numbers differed between populations but in our investigation, it did not appear that this was mediated by differences in microbiota composition. In mammals, the function of the immune system is intrinsically linked to the microbiota due to the immunomodulatory effects of a number of bacterial metabolites, such as, short-chain fatty acids^67^, bacterial polysaccharides^5^, and aryl hydrocarbon receptor ligands^68^. These metabolites are important regulators of haematopoiesis^69^, they promote immune cell emigration^70^, guide lymphoid organogenesis^5^, control inflammatory responses^67^, and directly inhibit pathogens^68^. There have been no investigations into the role that microbial metabolites play in reptilian immune development, but it is likely that similar relationships exist in all vertebrates, based on the highly conserved nature of immune function in animals^7^. Alterations in the relative abundance of bacterial phyla within the microbiota of mice have been associated with increased risk of inflammatory disease^67^, and deficits in innate immune cell populations^69^. Therefore, it is plausible that the relative increase in Firmicutes, may be associated with a decrease in circulating heterophils and basophils in animals from Port Hedland, due to inhibition of cell maturation, or egression from the bone marrow due to alterations in bacterial metabolite concentrations. This may have been more apparent if we had been able to measure some of these metabolomics, rather than relying solely on microbiota composition as a measure of immune function. The role that the microbiota plays in reptile immunity warrants further investigation and is an important step in understanding the vertebrate-microbiota relationship and its evolution.

We detected a difference in stress indicators between the two populations of turtles, with animals from Crab Island having a higher H:L ratio than animals from Port Hedland (p = 0.015, Table 1). Some researchers have shown that specific bacterial populations may exacerbate or dampen the stress responses in mice, and that it may be controlled with supplementation of probiotics^71^. However, in our data we did not find any correlation between microbiota and stress, and interpreting indicators of stress in wild animals is complex^72^. The observed differences in our study may be due to the presence of saltwater crocodiles (*Crocodylus porosus*) in the water and on the beaches of Crab Island, which are predators of both adult turtles and hatchlings and have been reported to occur over the nesting season on Crab Island^73^.

Haematology and biochemistry parameters in reptiles are dynamic, and change considerably in response to a variety of intrinsic and extrinsic factors such as age, sex, reproductive status, nutrition, climate, and season^20,30,74–77^. These changes are not necessarily representative of changes in health status, but are more reflective of the body’s response to altered physiological state. Therefore, it is fundamentally incorrect to infer health or illness, when making comparisons between individuals (or populations), across different life stages or under disparate environmental conditions. As a result, we have made no attempt to compare our results with non-nesting females, as it is expected that there would be considerable differences due to the increased physiological demands of vitellogeneis and fasting that accompanies egg production and laying in sea turtles. Additionally, blood results for non-nesting flatback turtles are non-existent, and we would only be able to make comparisons with other sea turtle species which is even less valid than between animals within the same species, but at different life stages.

In this investigation we show results that indicate that microbiota composition does not play a significant part in determining some of the blood parameters of flatback turtles. The role that specific OTUs play in modulating inflammation, immune maturation, protein and mineral metabolism, and overall health of reptiles is an area that requires further study. This research highlights the importance of interpreting both blood parameters and microbiota in the context of locality.

## Supplementary information

Supplementary Figure 1.

## Data Availability

All data not presented in this article or in the supplementary material have been submitted to The National Center for Biotechnology Information www.ncbi.nlm.nih.gov.

## References

[1] Lee YK, Mazmanian SK (2010). Has the microbiota played a critical role in the evolution of the adaptive immune system?. Science.

[2] McFall-Ngai M (2013). Animals in a bacterial world, a new imperative for the life sciences. Proc. Natl Acad. Sci. USA.

[3] Dethlefsen L, McFall-Ngai M, Relman DA (2007). An ecological and evolutionary perspective on human-microbe mutualism and disease. Nature.

[4] Schroeder BO, Backhed F (2016). Signals from the gut microbiota to distant organs in physiology and disease. Nat. Med..

[5] Mazmanian SK, Liu CH, Tzianabos AO, Kasper DL (2005). An immunomodulatory molecule of symbiotic bacteria directs maturation of the host immune system. Cell.

[6] Thaiss CA, Zmora N, Levy M, Elinav E (2016). The microbiome and innate immunity. Nature.

[7] Zimmerman LM, Vogel LA, Bowden RM (2010). Understanding the vertebrate immune system: insights from the reptilian perspective. J. Exp. Biol..

[8] Ahasan, M. S., Waltzek, T. B., Huerlimann, R. & Ariel, E. Fecal bacterial communities of wild-captured and stranded green turtles (Chelonia mydas) on the Great Barrier Reef. *FEMS Microbiol. Ecol*. **93**; 10.1093/femsec/fix139 (2017). 10.1093/femsec/fix139 29069420

[9] Abdelrhman KF (2016). A first insight into the gut microbiota of the sea turtle *Caretta caretta*. Front. Microbiol..

[10] Biagi E (2019). Faecal bacterial communities from Mediterranean loggerhead sea turtles (*Caretta caretta*). Environ. Microbiol. Rep..

[11] Scheelings, T. F. *The microbiota of sea turtles* PhD thesis, Monash University, (2019).

[12] Price JT (2017). Characterization of the juvenile green turtle (*Chelonia mydas*) microbiome throughout an ontogenetic shift from pelagic to neritic habitats. PLoS One.

[13] Campos P, Guivernau M, Prenafeta-Boldu FX, Cardona L (2018). Fast acquisition of a polysaccharide fermenting gut microbiome by juvenile green turtles *Chelonia mydas* after settlement in coastal habitats. Microbiome.

[14] Ahasan MS, Waltzek TB, Huerlimann R, Ariel E (2018). Comparative analysis of gut bacterial communities of green turtles (*Chelonia mydas*) pre-hospitalization and post-rehabilitation by high-throughput sequencing of bacterial 16S rRNA gene. Microbiol. Res..

[15] Arizza V (2019). New insights into the gut microbiome in loggerhead sea turtles Caretta caretta stranded on the Mediterranean coast. PLoS One.

[16] *The IUCN Red List of Threatened Species. Version 2019-1*., http://www.iucnredlist.org/details/4615/0 (2019).

[17] Limpus, C. J. A Biological Review of Australian Marine Turtle Species. 5. Flatback turtle, *Natator depressus* (Garman). 1–53 (Queensland Environmental Protection Agency, Queensland, 2008).

[18] Limpus, C. J., Gyuris, E. & Miller, J. D. Reassessment of the taxonomic status of the sea turtle genus *Natator* McCulloch, 1908, with a redescription of the genus and species. *Transactions of the Royal Society of South Australia* **112**, 1–9; ci.nii.ac.jp/naid/10009670726/en/ (1988).

[19] Dutton, P., Broderick, D. & FitzSimmons, N. In Western *Pacific Sea Turtle Cooperative Research & Management Workshop* (ed. Kinan, I.) 93–101 (Western Pacific Regional Fishery Management Council, Honolulu, 2002).

[20] Heatley, J. J. & Russell, K. E. Clinical chemistry. In *Mader’s Reptile Medicine and Surgery* 3rd Ed. (eds Divers, S. J. & Stahl, S. J.) 319–331 (Elsevier, 2019).

[21] Ferrer M, Dobado-Berrios P (1998). Factors affecting plasma chemistry values of the Spanish imperial Eagle, *Aquila adalberti*. Comp. Biochem. Physiol. A.

[22] Whiting SD, Guinea ML, Limpus CJ, Fomiatti K (2007). Blood chemistry reference values for two ecologically distinct populations of foraging green turtles, eastern Indian Ocean. Comp. Clin. Path..

[23] Poljičak-Milas N (2004). Serum biochemical values in fallow deer (*Dama dama* L.) from different habitats in Croatia. Eur. J. Wildl. Res..

[24] Eichmiller JJ, Hamilton MJ, Staley C, Sadowsky MJ, Sorensen PW (2016). Environment shapes the fecal microbiome of invasive carp species. Microbiome.

[25] Amato KR (2013). Habitat degradation impacts black howler monkey (*Alouatta pigra*) gastrointestinal microbiomes. ISME J..

[26] Ren T (2017). Seasonal, spatial, and maternal effects on gut microbiome in wild red squirrels. Microbiome.

[27] Bahrndorff S, Alemu T, Alemneh T, Lund Nielsen J (2016). The Microbiome of Animals: Implications for. Conservation Biology. Int. J. Genomics.

[28] Duchene S (2012). Marine turtle mitogenome phylogenetics and evolution. Mol. Phylogenetics Evol..

[29] Rafferty A, Evans R, Scheelings T, Reina R (2013). Limited oxygen availability in utero may constrain the evolution of live birth in reptiles. Am. Nat..

[30] Campbell, T. W. & Ellis, C. K. Hematology of reptiles. In *Avian and exotic animal hematology and cytology* 3rd Ed. (eds Campbell, T. W. & Ellis, C. K.) 3–82 (Blackwell Publishing, 2007).

[31] Dien, F. J., Wilson, A., Fischer, D. & Langenberg, P. Avian leucocyte counting using the hemocytometer. *J. Zoo Widl. Med*. **25**, 432–437; www-jstor-org.ezproxy.lib.monash.edu.au/stable/20095395 (1994).

[32] Davis AK, Maney DL, Maerz JC (2008). The use of leukocyte profiles to measure stress in vertebrates: a review for ecologists. Funct. Ecol..

[33] Green, M. R., Hughes, H., Sambrook, J. & MacCallum, P. Molecular cloning: a laboratory manual. In *Molecular cloning: A laboratory manual* 4th Ed. (eds Green, M. R. & Sambrook, J.) 44–47 (Cold Spring Harbour Laboratory Press, 2012).

[34] Fadrosh DW (2014). An improved dual-indexing approach for multiplexed 16S rRNA gene sequencing on the Illumina MiSeq platform. Microbiome.

[35] Caporaso JG (2010). QIIME allows analysis of high-throughput community sequencing data. Nat. Methods.

[36] Edgar RC (2010). Search and clustering orders of magnitude faster than BLAST. Bioinformatics.

[37] Ashelford KE, Chuzhanova NA, Fry JC, Jones AJ, Weightman AJ (2005). At least 1 in 20 16 S rRNA sequence records currently held in public repositories is estimated to contain substantial anomalies. Appl. Environ. Microbiol..

[38] DeSantis TZ (2006). Greengenes, a chimera-checked 16S rRNA gene database and workbench compatible with ARB. Appl. Environ. Microbiol..

[39] Altschul SF (1997). Gapped BLAST and PSI-BLAST: a new generation of protein database search programs. Nucleic Acids Res..

[40] Zakrzewski M (2017). Calypso: a user-friendly web-server for mining and visualizing microbiome-environment interactions. Bioinformatics.

[41] McMurdie PJ, Holmes S (2013). phyloseq: an R package for reproducible interactive analysis and graphics of microbiome census data. PLoS One.

[42] Clayton JB (2016). Captivity humanizes the primate microbiome. Proc. Natl Acad. Sci. USA.

[43] Cheng Y (2015). The Tasmanian devil microbiome-implications for conservation and management. Microbiome.

[44] Gibson KM (2019). Gut microbiome differences between wild and captive black rhinoceros - implications for rhino health. Sci. Rep..

[45] Kohl KD, Skopec MM, Dearing MD (2014). Captivity results in disparate loss of gut microbial diversity in closely related hosts. Conserv. Physiol..

[46] Schmidt E, Mykytczuk N, Schulte-Hostedde AI (2019). Effects of the captive and wild environment on diversity of the gut microbiome of deer mice (Peromyscus maniculatus). The ISME Journal.

[47] Borbon-Garcia A, Reyes A, Vives-Florez M, Caballero S (2017). Captivity shapes the gut microbiota of andean bears: insights into health surveillance. Front. Microbiol..

[48] Wang L (2019). Pere David’s deer gut microbiome changes across captive and translocated populations: implications for conservation. Evol. Appl..

[49] Delport TC, Power ML, Harcourt RG, Webster KN, Tetu SG (2016). Colony location and captivity influence the gut microbial community composition of the Australian sea lion (*Neophoca cinerea*). Appl. Environ. Microbiol..

[50] Kostic AD, Howitt MR, Garrett WS (2013). Exploring host-microbiota interactions in animal models and humans. Genes Dev..

[51] Gootenberg DB, Turnbaugh PJ (2011). Companion animals symposium: humanized animal models of the microbiome. J. Anim. Sci..

[52] Fedorov VB (2011). Modulation of gene expression in heart and liver of hibernating black bears (*Ursus americanus*). BMC Genomics.

[53] Otis JP, Sahoo D, Drover VA, Yen CL, Carey HV (2011). Cholesterol and lipoprotein dynamics in a hibernating mammal. PLoS One.

[54] Sommer F (2016). The gut microbiota modulates energy metabolism in the hibernating brown bear *Ursus arctos*. Cell Reports.

[55] Sayin, S. I. *et al*. Gut microbiota regulates bile acid metabolism by reducing the levels of tauro-beta-muricholic acid, a naturally occurring FXR antagonist. *Cell Metab*., 225–235; 10.1016/j.cmet.2013.01.003 (2013). 10.1016/j.cmet.2013.01.003 23395169

[56] Hays GC, Broderick AC, Glen F, Godley BJ (2002). Change in body mass associated with long-term fasting in a marine reptile: the case of green turtles (*Chelonia mydas*) at Ascension Island. Can. J. Zool..

[57] Martinez-Jimenez D, Hernandez-Divers SJ (2007). Emergency care of reptiles. Vet. Clin. North Am. Exot. Anim. Pract..

[58] Mach N, Fuster-Botella D (2017). Endurance exercise and gut microbiota: A review. J. Sport Health Sci..

[59] Lv LX (2016). Alterations and correlations of the gut microbiome, metabolism and immunity in patients with primary biliary cirrhosis. Environ. Microbiol..

[60] Pendoley KL (2014). Reproductive biology of the flatback turtle Natator depressus in Western Australia. Endangered Species Research.

[61] Zhao J, Zhang X, Liu H, Brown MA, Qiao S (2019). Dietary protein and gut microbiota composition and function. Curr. Protein Pept. Sci..

[62] Moyle V (1949). Nitrogenous excretion in chelonian reptiles. Biochem. J..

[63] Scheelings, T. F. Anatomy and physiology. In *BSAVA Manual of Reptiles* 3rd Ed. (eds Girling, S. & Raiti, P.) 1–25 (British Small Animal Veterinary Association, 2019).

[64] Yu Y, Liu Q, Li H, Wen C, He Z (2018). Alterations of the Gut Microbiome Associated With the Treatment of Hyperuricaemia in Male Rats. Front. Microbiol..

[65] Vaziri ND (2016). Effect of synbiotic therapy on gut-derived uremic toxins and the intestinal microbiome in patients with CKD. Clin. J. Am. Soc. Nephrol..

[66] Hernandez-Divers SJ (2004). Endoscopic renal evaluation and biopsy of chelonia. Vet. Rec..

[67] Trompette A (2014). Gut microbiota metabolism of dietary fiber influences allergic airway disease and hematopoiesis. Nat. Med..

[68] Rooks MG, Garrett WS (2016). Gut microbiota, metabolites and host immunity. Nat. Rev. Immunol..

[69] Khosravi A (2014). Gut microbiota promote hematopoiesis to control bacterial infection. Cell Host Microbe.

[70] Shi C (2011). Bone marrow mesenchymal stem and progenitor cells induce monocyte emigration in response to circulating Toll-like receptor ligands. Immunity.

[71] Dinan TG, Cryan JF (2012). Regulation of the stress response by the gut microbiota: implications for psychoneuroendocrinology. Psychoneuroendocrinology.

[72] Johnstone CP, Reina RD, Lill A (2012). Interpreting indices of physiological stress in free-living vertebrates: a review. J. Comp. Physiol. B.

[73] Sutherland RW, Sutherland EG (2003). Status of the flatback sea turtle (*Natator depressus*) rookery on Crab Island, Australia, with notes on predation by crocodiles. Chelonian Conserv. Bi..

[74] Scheelings TF, Rafferty AR (2012). Hematologic and serum biochemical values of gravid freshwater Australian chelonians. J. Wildl. Dis..

[75] Scheelings TF, Williamson SA, Reina RD (2016). Hematology and serum biochemistry for free-ranging freshwater crocodiles (*Crocodylus johnstoni*) in Western Australia. J. Wildl. Dis..

[76] Scheelings T, Jessop T (2011). Influence of capture method, habitat quality and individual traits on blood parameters of free-ranging lace monitors (*Varanus varius*). Aust. Vet. J..

[77] Rafferty AR, Scheelings TF, Foley LJ, Johnstone CP, Reina RD (2014). Reproductive investment compromises maternal health in three species of freshwater turtle. Physiological and Biochemical Zoology.

