# Supplementary material for: No correlation between microbiota composition and blood parameters in nesting flatback turtles (Natator depressus)
Source: Sci Rep. 2020 May 20;10:8333. doi: 10.1038/s41598-020-65321-5 (PMC7239873; doi:10.1038/s41598-020-65321-5)
Supplement: Supplementary file 1 — Supplementary Figure 1. [file 41598_2020_65321_MOESM1_ESM.docx]

**No correlations between microbiota composition and blood parameters in nesting flatback turtles (*Natator depressus*)**

T. Franciscus Scheelings^1*^, Robert J. Moore^2^, Thi Thu Hao Van^2^, Marcel Klaassen^3^ & Richard D. Reina^1^

**^1^***School of Biological Sciences, Monash University, Wellington Rd, Clayton, Victoria, 3800, Australia*

^2^*School of Science, RMIT University, Bundoora West Campus, Plenty Rd, Bundoora, Victoria, 3083, Australia*

^3^*Centre for Integrative Ecology, Deakin University, Waurn Ponds, Victoria, 3216, Australia*

*Corresponding author: email [fscheelings@hotmail.com](mailto:fscheelings@hotmail.com), Ph: +61 425797392

**Supplementary Material**

**Supplementary Figure 1.** Graphical representations (scatterplots) of correlations between microbiota composition and blood analytes. No significant correlations were found for any of the blood parameters and microbiota composition for the animals in our study.

| 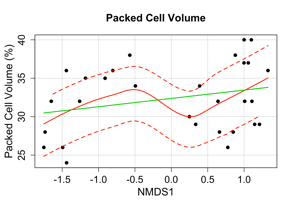 | 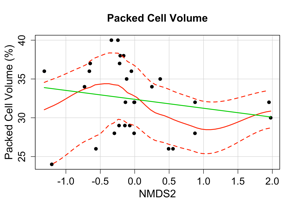 |
| --- | --- |
| 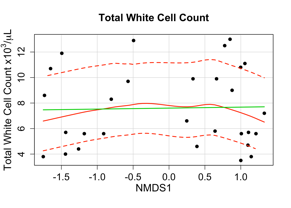 | 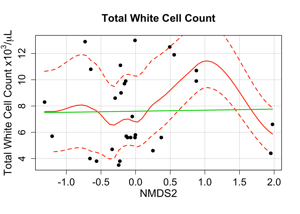 |
| 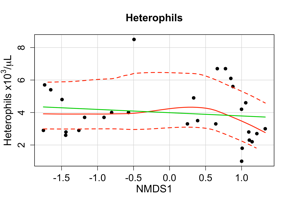 | 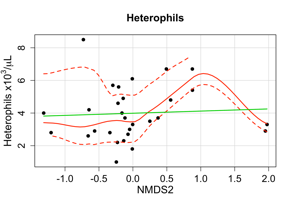 |
| 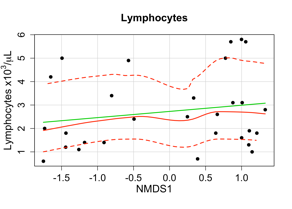 | 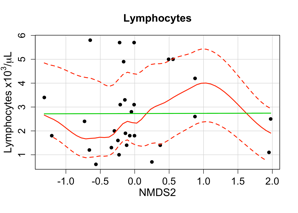 |
| 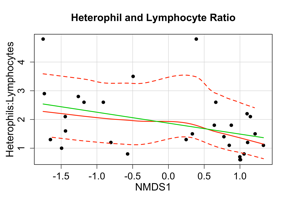 | 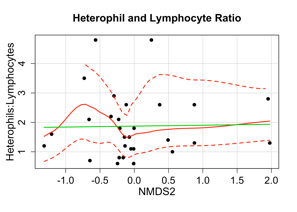 |
| 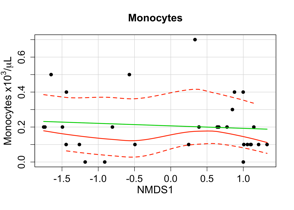 | 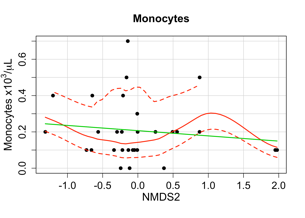 |
| 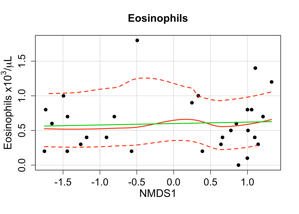 | 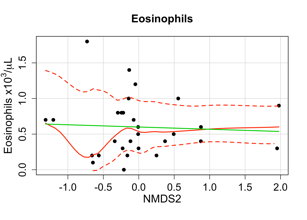 |
| 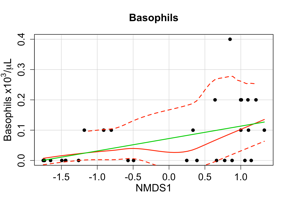 | 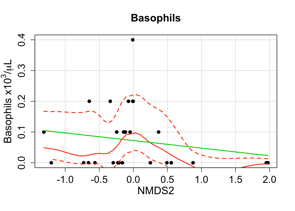 |
| 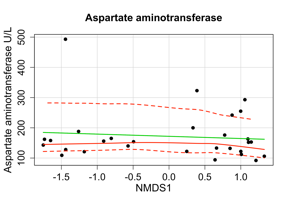 | 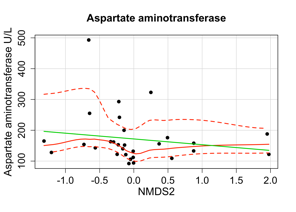 |
| 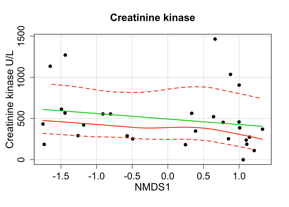 | 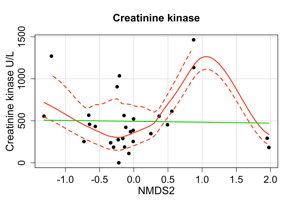 |
| 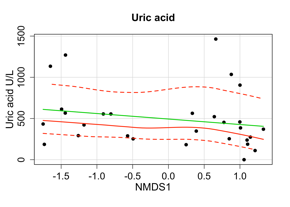 | 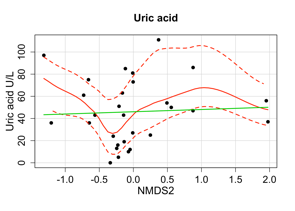 |
| 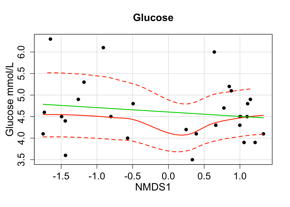 | 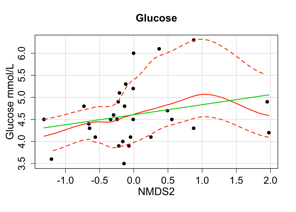 |
| 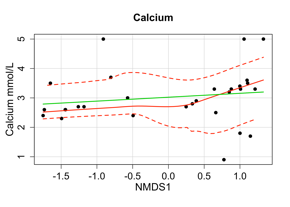 | 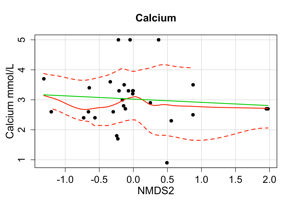 |
| 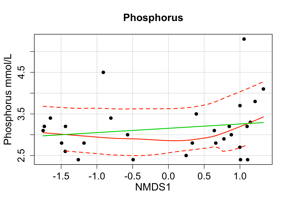 | 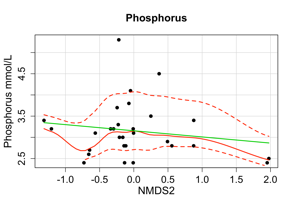 |
| 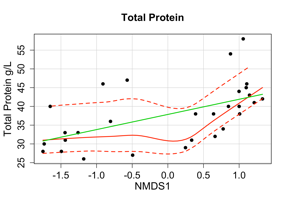 | 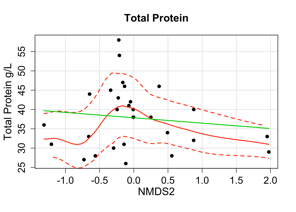 |
| 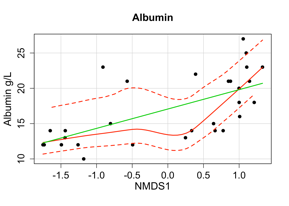 | 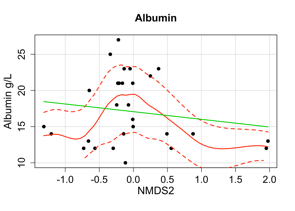 |
| 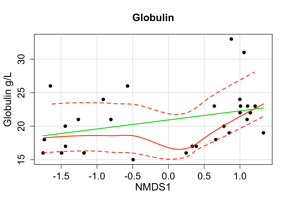 | 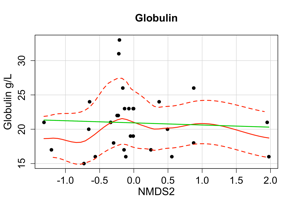 |
| 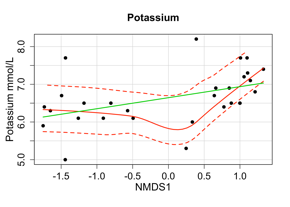 | 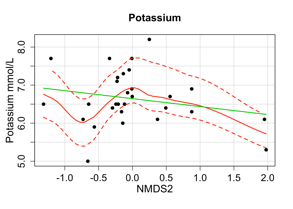 |
| 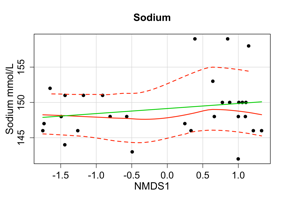 | 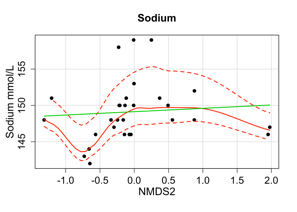 |
